# The role of magnetic resonance imaging in the preoperative evaluation of anal fistulas

**DOI:** 10.1038/s41598-019-54441-2

**Published:** 2019-11-29

**Authors:** Duc Vo, Chien Phan, Linh Nguyen, Huyen Le, Tin Nguyen, Hung Pham

**Affiliations:** 1 Department of Diagnostic Imaging, University Medical Center, Ho Chi Minh City, Vietnam; 2 Department of Proctology, University Medical Center, Ho Chi Minh City, Vietnam; 3 Adelaide Medical School, The University of Adelaide, Adelaide, SA Australia

**Keywords:** Gastrointestinal diseases, Anal diseases

## Abstract

This study aimed to determine the role of magnetic resonance imaging (MRI) in diagnosing and describing the characteristics of fistula-in-ano, and the agreement between MRI and operative findings. We conducted a retrospective study in 367 patients with fistula-in-ano who were diagnosed and had an operation at the University Medical Center between January 2016 and January 2018. MRI findings were evaluated and compared with surgical findings using the kappa coefficient (k) method. 367 patients (327 male and 40 female, mean age 39.3 ± 12.4 years). A total of 411 primary fistulas were found during surgery. There was a strong agreement between MRI and surgery for classifying primary tracts (k = 0.89) and detecting secondary tracts (k = 0.94). While the sensitivity and specificity of MRI for detecting internal openings were 99% and 85.2% respectively; these rates were 100% for abscesses. Both T2-weighted turbo spin-echo (T2W TSE) and postcontrast fat-saturated T1-weighted turbo spin-echo (FS T1W TSE) sequences showed high sensitivity (96.6% and 98.4% respectively) and specificity (92.6% and 81.5% respectively) for depicting internal openings and secondary tracts. Post-contrast FS T1W TSE sequence was very effective in detecting abscesses with an accuracy of 100%. In conclusion, MRI can be considered an accurate tool for the preoperative evaluation of fistula-in-ano, which is a major determinant of the surgical outcome. Both T2W TSE and post-contrast FS T1W TSE sequences are highly accurate in depicting the features of fistula-in-ano. If there are no contraindications, contrast administration is recommended to differentiate abscesses from active inflammation.

## Introduction

Fistula-in-ano is an inflammatory disorder of anorectal region characterised by a tract between the anal canal and the perianal skin^1,2^. Fistula-in-ano is usually a sequela of a poorly managed perianal abscess. This condition can also be associated with tuberculosis, cancer, and radiotherapy, etc.^2,3^.

Fistula-in-ano is the second most common anorectal disease after haemorrhoids^2^. Surgery is considered the treatment of choice aiming to avoid recurrence and preserve anal sphincter function. The risk of recurrence increases to 25% if surgeons fail to recognise and remove radically a fistula and its associated elements during corrective surgery, especially internal openings and secondary tracts^1,4–6^. Accordingly, a precise and comprehensive preoperative assessment of fistula tract is a pivotal diagnostic strategy and contributes significantly to the success rate of surgery.

Before the era of magnetic resonance imaging (MRI), fistulography was used to evaluate fistula-in-ano. However, this technique has a low diagnostic accuracy (~16%), and inability to visualise secondary tracts, abscesses and the sphincter complex due to its suboptimal contrast opacification^7^. As a result, fistulograms are not able to provide information about the relationship between fistula tracts and anal sphincters. Endoanal ultrasonography is the first imaging technique that provides the anatomical details of anal canal^1^. It can be used for the diagnosis and management of not only abscesses and fistula-in-ano, but also anorectal and prostate tumours. Endoanal ultrasonography is particularly helpful in identifying primary fistulous tracts and internal openings with high accuracy rates^8,9^. However, the limited field of view is regarded as an inherent limitation of this technique, discounting its value to evaluate secondary tracts or supralevator extensions of a primary tract.

Recently, MRI has been considered the ‘gold standard’ technique for the preoperative evaluation of fistula-in-ano. An accurate and comprehensive assessment to detect primary tracks, associated ramifications and abscesses plays a crucial role in determining surgical outcomes and minimising complications, such as faecal incontinence, as well as recurrent lesions^1,10–12^.

The aim of this study was to evaluate the accuracy of MRI for the diagnosis and characterisation of fistula-in-ano and the agreement between preoperative MRI and surgical findings.

## Materials and Methods

This retrospective study was conducted in the University Medical Center, Ho Chi Minh City. The protocol was reviewed and approved by the Human Research Ethics Committee of the University Medical Center of Ho Chi Minh City. The study was performed in accordance with the Declaration of Helsinki. All the patients, or their guardians, provided written informed consent prior to their involvement in the study.

### Subjects

The images of patients who had preoperative MRI assessment and surgery for fistula-in-ano from January 1 2016 to January 31 2018 were collected. All patients also underwent a physical examination by a proctologist to document the number and location of cutaneous openings after detailed medical history had been collected.

### MRI protocol

MRI examinations were performed on either 1.5 T (Magnetom Avanto, Siemens Healthcare Limited, Germany) or 3.0 T MR (Magnetom, Siemens Healthcare Limited, Germany) scanners using a phased-array surface coil with 6 channels.

No patient preparation was required and patients were placed flat on their back. Precontrast images obtained were as follows: sagittal T2-weighted turbo spin-echo (T2W TSE), oblique coronal fat-saturated (FS) T2W TSE, oblique axial T2W TSE, oblique axial FS T2W TSE, oblique axial T1-weighted turbo spin-echo (T1W TSE) and axial FS T1W TSE. After an intravenous administration of gadolinium (0.2 ml/kg), post-contrast FS T1W TSE images were acquired in three planes (sagittal, coronal and axial). Parameters of the used sequences are shown in Table 1.

**Table 1:** MRI sequences for preoperative assessment of fistula-in-ano.

Sequence		TR (ms)	TE (ms)	FOV (cm)	Matrix	Thick (mm)	Gap (mm)	NSA
T2W TSE sagittal		4570	86	23	320 × 256	3,5	0,35	1
T2W TSE axial		5000	86	20	320 × 240	3,5	0,35	2
FS T2W TSE axial		5160	86	20	320 × 240	3,5	0,35	2
FS T2W TSE coronal		3220	74	25	320 × 240	3,2	0,32	1
T1W TSE axial		544	10	20	320 × 224	3,5	0,35	1
FS T1W TSE axial		670	10	20	320 × 224	3,5	0,35	1
Postcontrast FS T1W TSE	axial	670	10	20	320 × 224	3,5	0,35	2
	coronal	600	12	25	320 × 256	3,2	0,32	1
	sagittal	655	12	23	320 × 224	3,5	0,35	1

TSE: turbo spin echo, FS: fat-saturated, TR: repetition time, TE: echo time, FOV: field of view, NSA: number of acquisitions.

### Image analysis

Images were interpreted and reported independently on picture archiving and communication system (PACS) by two radiologists who had more than 5 years of experience in analysing fistula-in-ano MRI. In the cases where there were discrepancies in interpretation between the two radiologists, a senior radiologist’s evaluation was considered the final result.

The following characteristics were assessed for each fistula-in-ano: the location of primary tracts, the presence of secondary tracts and abscess formation and the site of internal and external openings. Fistulas were classified according to the Parks and St. James’s University Hospital classifications^13,14^. In the image interpretation, it was assumed that a fluid collection larger than 10 mm in diameter with rim enhancement on post-contrast T1W TSE images was an abscess as per the criteria of Singh *et al*. and Torkzad *et al*.^15,16^.

During surgery, the characteristics of each fistula-in-ano were also carefully documented and then used as a reference standard to compare to MRI findings.

### Statistical analysis

For each MRI characteristic, 2 × 2 contingency table was used to calculate sensitivity, specificity, positive predictive value (PPV), negative predictive value (NPV), and diagnostic accuracy. Agreement between the MRI and surgical findings was assessed using the weighted kappa coefficient (k) with a 95% confidence interval. The degree of agreement was classified as follows: poor (k < 0.2), fair (0.2 ≤ k < 0.4), moderate (0.4 ≤ k < 0.6), good (0.6 ≤ k < 0.8), or very good (k ≥ 0.8). All analyses were performed using STATA version 14 (STATA Corp., Texas, USA). A P value < 0.05 was considered significant in all analyses. Data are shown as mean values ± SDs.

## Results

367 patients were eligible for the study. They were 320 males and 47 females with a ratio of 9:1. Their ages ranged from 12 to 84 years with a mean of 39.3 ± 12.4 years. Of 367 patients, 6.0% were aged over 60 years, 91.6% were aged from 20 to 60 years and only 2.4% were aged less than 20 years. 27% of patients had previous surgery for anal fistula.

### External opening

The external openings were identified in 353/367 patients (96.2%) with 442 external openings. 289 (81.9%) patients had one external opening. 64 (18.1%) patients had multiple external openings: 47 (73.4%) had two, 10 (15.6%) had three, 6 (9.4%) had four and 1 (1.6%) had five external openings.

The mean distance between external opening and anal verge was 2.9 ± 2 cm. Distances of different fistula types are shown in Table 2. 95% of external openings were within 5 cm from the anal verge and 60.4% of external openings were posterior.

**Table 2:** Distance between external opening and anal verge.

	≤3 cm	3–5 cm	>5 cm
Intersphincteric fistula	88 (95.7%)	4 (4.3%)	0 (0%)
Low transsphincteric fistula	150 (71.8%)	46 (22.0%)	13 (6.2%)
High transsphincteric fistula	5 (9.3%)	45 (83.3%)	4 (7.4%)
Supra and extrasphincteric fistula	0 (0%)	10 (83.3%)	2 (16.7%)

### Primary tract

411 primary tracts were detected in 367 (%) patients during surgery. 263 (64.0%) tracts were transsphincteric, 92 (22.4%) tracts were intersphincteric according to the Parks classification (Table 3). Using this classification system, surgeons also reported 7 (1.7%) superficial fistulas and 37 (9.0%) blind tracts, which were left unclassified. Up to 333 (90.7%) patients had a single primary tract and 3 (0.8%) patients had four primary tracts.

**Table 3:** Agreement between MRI and surgery in the classification of primary tract according to the Parks classification.

Surgery	Inter	Trans	Supra	Extra	Superficial	Blind tract	No primary tract	Total
MRI								
Inter	92	16	0	0	0	4	1	113
Trans	0	245	0	0	0	0	0	245
Supra	0	0	3	0	0	0	0	3
Extra	0	0	0	9	0	0	0	9
Superficial	0	0	0	0	7	0	0	7
Blind tract	0	0	0	0	0	32	0	32
No primary tract	0	2	0	0	0	1	0	3
Total	92	263	3	9	7	37	1	412

Inter: Intersphincteric fistula, Trans: transsphincteric fistula, Supra: suprasphincteric fistula, Extra: extrasphincteric fistula, Superficial: superficial fistula.

Data are presented as numbers of fistulas. Kappa value: 0.89 (0.85–0.94), p < 0.001.

According to the St. James’s University Hospital classification, 89 (24.1%) patients had grade 1, 24 (6.5%) patients had grade 2, 157 (42.4%) patients had grade 3, 88 (23.8%) patients had grade 4, and 12 (3.2%) patients had grade 5 fistulas (Table 4).

**Table 4:** MRI grading of anal fistulas according to St. James’s University Hospital classification.

Grade	Number
Grade 1	89 (24.1%)
Grade 2	24 (6.5%)
Grade 3	157 (42.4%)
Grade 4	88 (23.8%)
Grade 5	12 (3.2%)

Low transsphincteric fistulas occurred more frequently (79.5%) than high transsphincteric fistulas (20.5%). There was a strong agreement for primary tract classification between MRI and surgical findings, with k = 0.89 (Table 3).

### Internal opening

385 internal openings were detected in 367 patients. 360 (93.5%) internal openings were located at the dentate line with a mean distance to the anal verge of 2.2 ± 0.2 cm and 179 (46.5%) internal openings were located at 6 o’clock position.

MRI correctly identified 381/385 (99.0%) internal openings. In one case, MRI failed to detect the presence of internal openings, and in 3 other cases, there was a mismatch between MRI and surgical findings.

### Abscess

47 abscesses were detected in 41 (11.2%) patients. Abscess locations were as follow: 16 (34%) in ischioanal, 16 (34%) in intersphincteric, 10 (21.3%) in perianal, 4 (8.5%) in supralevator and 1 (2.1%) in deep postanal space. 15/41 (36.6%) patients had horseshoe abscess development.

The mean diameter of abscesses was 2.3 ± 1.1 cm. Both sensitivity and specificity of MRI in diagnosing abscesses were 100%.

### Secondary tract

132 secondary tracts were detected on MRI in 101 (27.5%) patients. 78 (77.2%) patients had one secondary tract. 23 (22.8%) patients had multiple secondary tracts: 18 (17.8%) had two, 3 (3.0%) had three, 1 (1.0%) had four and 1 (1.0%) had five secondary tracts. The agreement between MRI and surgical findings in identifying the location of secondary tracts is shown in Table 5.

**Table 5:** Agreement between MRI and surgery for detecting the location of secondary tracts.

MRI	Surgery	Perianal	Inter	Ischio	Supra	Deep postanal	Sub	No secondary tract	Total
Perianal		3							3
Inter			45					6	51
Ischio				56				3	59
Supra					14			2	16
Deep postanal						3			3
Sub							0		0
No secondary tract			1				1	266	268
Total		3	46	56	14	3	1	277	400

Perianal: perianal space, Inter: Intersphincteric space, Ischio: ischioanal space, Supra: supralevator space, Deep postanal: deep postanal space, Sub: submucosa space.

Data are presented as numbers of secondary tracts. Kappa value: 0.94 (0.90–0.97), p < 0.001.

The sensitivity, specificity, PPV, NPV and accuracy of T2W TSE and post-contrast FS T1W TSE sequences to detect internal openings, secondary tracts and abscesses are shown in Table 6.

**Table 6:** Comparison between T2W TSE and post-contrast FS T1W TSE sequences in the characterisation of fistulas.

	Sequence	Sensitivity	Specificity	PPV	NPV	Accuracy
Internal opening	T2W TSE	96.6	92.6	99.5	65.8	96.4
	Postcontrast FS T1W TSE	98.4	81.5	98.7	78.6	97.3
Abscess	T2W TSE	not calculated	not calculated	not calculated	not calculated	not calculated
	Postcontrast FS T1W TSE	100	100	100	100	100
Secondary tract	T2W TSE	96.7	99.2	98.3	98.5	98.5
	Postcontrast FS T1W TSE	98.4	97.7	95.3	99.2	97.9

## Discussion

Our study has demonstrated that perianal fistula occurs predominantly in adult males with the male to female ratio of 9:1 and the mean age of disease is 40 years, which are consistent with previous reports^3,15,17,18^.

**Figure 1:**
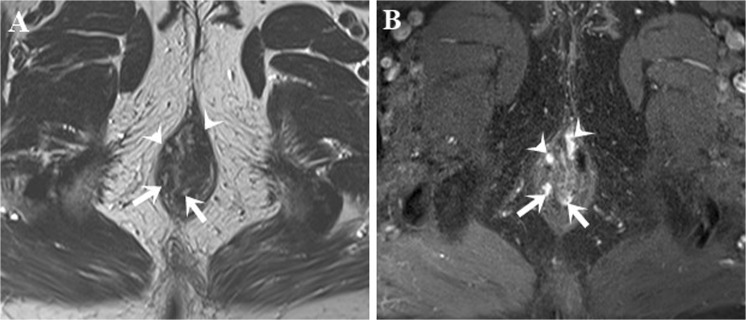
Intersphincteric and transsphincteric fistulas. Axial T2W TSE (**A**) and post-contrast FS T1W TSE (**B**) images show four fistulas in one patient: two intersphincteric fistulas (arrow) and two low transsphincteric fistulas penetrating the subcutaneous part of external anal sphincter (head arrow).

We observed a strong association between the distance from external opening to anal verge and the type of anal fistula. The majority of intersphincteric (95.7%) and low transsphincteric (71.8%) fistulas had their external openings near the anal verge (≤ 3 cm) (Fig. 1), while external openings were located more distally to the anal verge (> 3 cm) for the most of transsphincteric (90.7%), and all the suprasphincteric (100%) and extrasphincteric (100%) fistulas. However, there were some exceptional cases, especially when external openings on the posterior midline of scrotum. In these cases, external openings were distal to the anal verge, but fistulas might still be low-transsphincteric due to their long subcutaneous course.

MRI has been studied by many researchers and it is now recognised as an essential imaging modality in the preoperative evaluation and management of perianal fistulas. In particularly, MRI has the ability to detail accurately fistulas, associated abscesses and secondary extensions, which would otherwise be challenging for other radiology modalities. In addition, it can provide comprehensive images of the anatomical correlation between fistulas and anal sphincters, pelvic floor and levator ani muscle. This information is very important in surgical planning to remove completely infected lesions and reduce complications as well as recurrence. One previous study has demonstrated that the recurrence rate reduces substantially (~75%) following MRI navigated surgery^5^.

Consistent with the previous study by Buchanan *et al*.^8^, the majority of 411 primary tracts found during surgery were transsphincteric (64.0%) and intersphincteric (22.4%). These rates differ from the previous report that the most common fistulas are the intersphincteric^3,13^. However, this could be explained by our different study population which included only patients who had preoperative MRI prior to corrective surgery. In contrast to our study, other authors^3,13^ included those who had corrective surgery with or without preoperative MRI. Most of the patients in our study had a single primary tract (90.7%), which was similar to the previous study by Baik *et al*. (83%)^19^.

The strong agreement observed in our study (k = 0.89) between MRI and surgical findings in terms of the classification of primary tracts is very close to a previous study (k = 0.84)^8^. Similarly, Singh *et al*.^15^ and Beets-Tan *et al*.^20^ reported that MRI had a high accuracy rate (94% and 93% respectively) in detecting primary tracts. Our study, as well as previous studies, support the concept that MRI is the imaging modality of choice in the preoperative assessment of anal fistulas^8,15,16^. In our study, MRI was found to misclassify 10% of primary tracts compared with the reference standard. However, this misclassification rate could be attributed to a minor difference in the tract description between radiologists and surgeons. In particular, tracts crossing the most distal fibres of subcutaneous external sphincter were identified as intersphincteric fistulas on MRI, and then reassigned as transsphincteric fistulas by surgeons.

Precise evaluation of the location of internal openings is essential for a successful surgical outcome. On MRI, an internal opening is defined as the nearest point of a fistula to the anal canal, which is often seen in the intersphincteric space and it is nearly impossible to trace along a tract to its very end into the anal mucosa. Previous authors have suggested that the potential location of internal opening, in most cases, is often positioned in the most inflammatory area of intersphincteric space^5,20^. In our study, we used the similar strategy and noticed that the sensitivity, specificity, PPV, NPV and accuracy of MRI for depicting the internal opening were 99%, 85.2%, 99%, 85.2%, 98%, respectively. These findings are not surprising and in agreement with previous studies. Comparable high rates were reported by Beets-Tan *et al*.^20^ (sensitivity, specificity, PPV and NPV − 96%, 90%, 90% and 96% respectively) and Singh *et al*.^15^ (sensitivity, specificity and PPV − 96%, 80%, and 98% respectively). In another study^18^, the concordance rate for the identification of internal openings between MRI and surgery was 87%. The majority of internal openings were observed at the dentate line where most anal glands empty into the anus.

**Figure 2:**
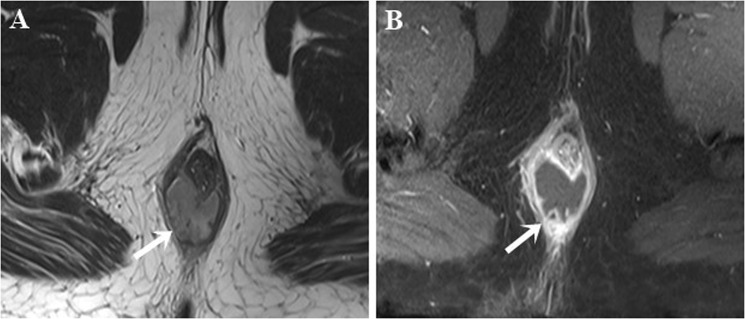
Intersphincteric horseshoe abscess. Axial T2W TSE (**A**) and post-contrast FS T1W TSE (**B**) images show a horseshoe abscess (arrow) with high signal intensity on T2W TSE and rim enhancement on post-contrast FS T1W TSE.

MRI has the advantages of resolution and large field of view, especially when a multichannel phased array coil is combined with a high field strength of 1.5-Tesla or 3-Tesla. In this study, we used the diameter-based criteria, proposed by Torkzad *et al*.^16^, to classify fluid collections, i.e., a roundish fluid collection ≥ 10 mm was identified as an abscess while an elongated fluid collection < 10 mm was identified as a fistula (Fig. 2). We have found that both sensitivity and specificity of MRI in differentiating abscesses from fistulas were 100%. Prior to our study, Beets-Tan *et al*.^20^ and Singh *et al*.^15^ also reported high sensitivity (96% and 87.5% respectively) and high specificity (97% and 95.2% respectively) with regards to abscess detection.

**Figure 3:**
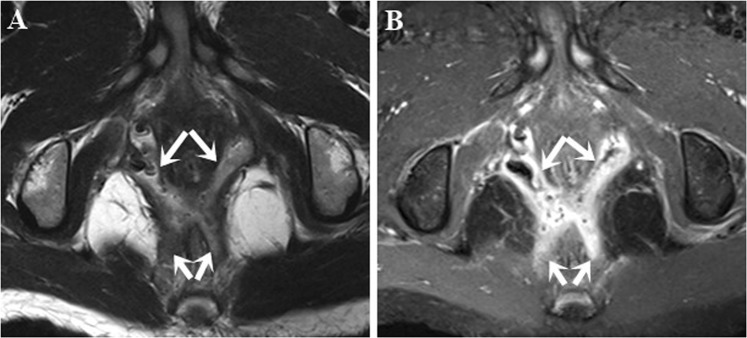
Secondary tracts. Axial T2W TSE (**A**) and post-contrast FS T1W TSE (**B**) images show a high transsphincteric fistula with secondary tracts in the ischioanal space (arrows).

The agreement between MRI and surgical findings in terms of identifying the location of secondary tracts was found to be good (k = 0.94) (Fig. 3), approximating to the previous study by Buchanan *et al*. (k = 0.88)^8^. There were 11 false-positive secondary tracts, identified as tiny or short fistulas on MRI. We suspect that these tracts were probably resected together with primary tracts, however, not detected by surgeons.

**Figure 4:**
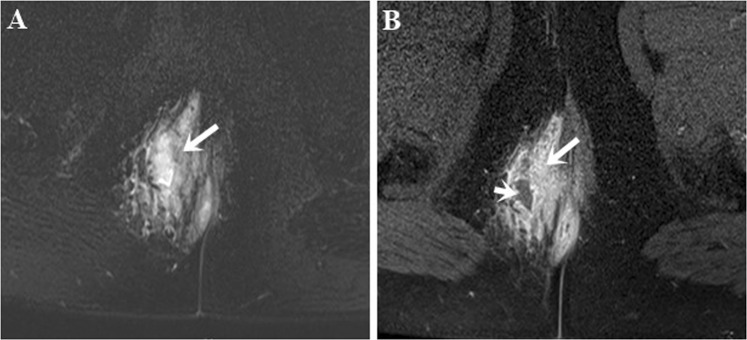
Abscess and active inflammation. Axial FS T2W TSE image (**A**) shows a hyperintense lesion which may be an abscess and/or active inflammation. Axial post-contrast FS T1W TSE image (**B**) differentiates an abscess (short arrow) from adjacent active inflammation (long arrow).

Both T2W TSE and post-contrast FS T1W TSE sequences demonstrated a high degree of accuracy in depicting the characteristics of fistula. In contrast to a ring-enhancing abscess, homogeneous enhancement is often seen in active inflammation on post-contrast FS T1W sequence (Fig. 4). Therefore, post-contrast FS T1W TSE sequence is suited better for distinguishing between an abscess and inflammation as they both appear hyperintense on T2W TSE sequences. However, T2W TSE sequences tended to be more optimal to position fistulas in their anatomical plane and assess their connection to anal sphincters. Torkzad *et al*.^16^ have indicated that T2W sequences must be included in the MRI protocol for the most reliable evaluation of fistula-in-ano in conjunction with post-contrast T1W sequences. Conversely, Singh *et al*.^15^ concluded that both T2W TSE and post-contrast FS T1W TSE sequences were comparable for assessing abscess and horseshoeing. However, a limitation of this study was that the study population did not include patients who had recurrent fistulas and/or previous surgery.

In interpreting our observations, specific limitations should be acknowledged: our study was retrospective in design; and all surgeons, albeit proctologists, had no equivalent level of experience in assessing fistula-in-ano, which might compromise the quality and consistency of the reference standard.

## Conclusions

Our study reinforces the importance of MRI in the characterisation and preoperative mapping of fistula-in-ano which are major contributors to the surgical prognosis. Using both T2W TSE and post-contrast FS T1W TSE sequences is a necessity for an adequate assessment of fistula-in-ano and contrast study should be included to differentiate an abscess from active inflammation.
